# Surveillance for Lyme Disease After Implementation of a Revised Case Definition — United States, 2022

**DOI:** 10.15585/mmwr.mm7306a1

**Published:** 2024-02-15

**Authors:** Kiersten J. Kugeler, Austin Earley, Paul S. Mead, Alison F Hinckley

**Affiliations:** 1 Division of Vector-Borne Diseases, National Center for Emerging and Zoonotic Infectious Diseases, CDC.

**Summary**

**What is already known about this topic?**

Lyme disease is the most common vectorborne disease in the United States, but risk is geographically focal. After the implementation of a revised surveillance case definition in 2022, high-incidence jurisdictions report cases based on laboratory evidence alone, without the need for case investigation to obtain clinical information.

**What is added by this report?**

In 2022, reported case counts were 1.7 times the annual U.S. average during 2017–2019. The relative change in incidence in 2022 increased with patient age.

**What are the implications for public health practice?**

Increase in Lyme disease cases in 2022 likely reflects changes in surveillance methods rather than change in disease risk. The case definition change improves standardization of surveillance across jurisdictions but precludes detailed comparison with historical data.

## Abstract

Lyme disease, a tickborne zoonosis caused by certain species of *Borrelia* spirochetes, is the most common vectorborne disease in the United States. Approximately 90% of all cases are reported from 15 high-incidence jurisdictions in the Northeast, mid-Atlantic, and upper-Midwest regions. After the implementation of a revised surveillance case definition in 2022, high-incidence jurisdictions report cases based on laboratory evidence alone, without need for additional clinical information. In 2022, 62,551 Lyme disease cases were reported to CDC, 1.7 times the annual average of 37,118 cases reported during 2017–2019. Annual incidence increased most in older age groups, with incidence among adults aged ≥65 years approximately double that during 2017–2019. The sharp increase in reported Lyme disease cases in 2022 likely reflects changes in surveillance methods rather than change in disease risk. Although these changes improve standardization of surveillance across jurisdictions, they preclude detailed comparison with historical data.

## Introduction

Lyme disease is a tickborne infection caused by spirochetes in the *Borrelia burgdorferi* sensu lato complex (*1*,*2*). Signs and symptoms of early disease include erythema migrans, a red, expanding rash often with central clearing, as well as fever and fatigue. Untreated infection can disseminate, affecting the heart, joints, and nervous system (*1*). National surveillance for Lyme disease in the United States began in 1991 and has documented a steady increase in incidence and geographic range. A majority of cases of Lyme disease are reported from 15 high-incidence jurisdictions (those reporting at least 10 confirmed cases per 100,000 population for 3 years) located in the Northeast, mid-Atlantic, and upper-Midwest regions* (*3*). Laboratory diagnosis relies almost exclusively on serologic testing for antibodies to *B. burgdorferi* using a two-tier process (*1*).

Before 2022, national surveillance for Lyme disease required the collection of clinical information, most often coupled with laboratory evidence of infection, to classify cases. As the number of Lyme disease infections has increased, the workload associated with collecting clinical information has proven prohibitive in several high-incidence jurisdictions, leading to the adoption of modified, jurisdiction-specific surveillance practices, including in New York and Massachusetts (*2*, *4*–*6*). These divergent approaches often precluded the reporting of cases to CDC and prevented accurate comparison of trends across jurisdictions and over time (*3*,*7*).

To address this challenge, effective January 1, 2022, the Council of State and Territorial Epidemiologists (CSTE), in partnership with CDC, revised the national surveillance case definition for Lyme disease.^†^ The revised case definition provides for reporting of cases from high-incidence jurisdictions based on laboratory evidence alone, without the need to collect additional clinical information. Cases reported from low-incidence jurisdictions still require supporting clinical information, although probable case classification criteria have been updated to only include those patients with objective signs of infection. This report summarizes the first year of Lyme disease surveillance data collected using the 2022 case definition and compares these data to cases reported during 2017–2019.

## Methods

Lyme disease cases are classified by state and local health departments according to CSTE surveillance case definitions and reported to CDC through the Nationally Notifiable Diseases Surveillance System.^§^ Because of reporting anomalies related to the COVID-19 pandemic (2020–2021) (*8*), cases reported in 2022 were compared with those reported during 2017–2019. 2020 U.S. Census Bureau data were used as population denominators for incidence calculations.^¶^ Several reporting dates were used to compare trends in seasonality. For the years 2017–2019, illness onset date was used, whereas for 2022, illness onset date, diagnosis date, laboratory test date, and date of laboratory report to health department were used. Data were analyzed using SAS software (version 9.4; SAS Institute). This activity was reviewed by CDC, deemed not research, and was conducted consistent with applicable federal law and CDC policy.**

## Results

### Overall: 2022 Versus 2017–2019

After implementation of a revised Lyme disease case definition, a total of 62,551 Lyme disease cases were reported to CDC in 2022 (including 59,734 from high-incidence jurisdictions and 2,817 from low-incidence jurisdictions).^††^ This finding represented an overall 68.5% increase from the annual average of 37,118 cases reported during 2017–2019, including a 72.9% increase in high-incidence jurisdictions and a 10.0% increase in low-incidence jurisdictions (Table). During 2022, 95.5% of reported cases were reported from high-incidence jurisdictions, compared with an average of 93.1% during 2017–2019. Lyme disease incidence in 2022 (18.9 cases per 100,000 population) was 68.8% higher than that during 2017–2019 (11.2). In 2022, median incidence among high-incidence jurisdictions (68.3 cases per 100,000) was 58% higher than that during 2017–2019 (43.3), although median incidence among low-incidence jurisdictions (0.52 cases per 100,000) was 24% lower than during 2017–2019 (0.68). 

**TABLE:** Number of reported Lyme disease cases and Lyme disease incidence, by jurisdiction and incidence category* — United States, 2017–2019 and 2022

Jurisdiction	No. of reported cases^†^			Incidence^§^		
	2017–2019^¶^	2022	Percent change**	2017–2019^¶^	2022	Incidence difference^††^
High-incidence jurisdictions*						
Connecticut	1,714	2,022	18.0	47.5	56.1	8.5
Delaware	590	298	−49.5	59.6	30.1	−29.5
District of Columbia	88	77	−12.5	12.7	11.2	−1.5
Maine	1,807	2,653	46.8	132.7	194.7	62.1
Maryland	1,563	2,035	30.2	25.3	32.9	7.6
Massachusetts	144	5,052	3,408.3	2.1	71.9	69.8
Minnesota	1,796	2,685	49.5	31.5	47.1	15.6
New Hampshire	1,506	1,085	−28.0	109.4	78.8	−30.6
New Jersey	4,237	5,897	39.2	45.6	63.5	17.9
New York	4,345	16,798	286.6	21.5	83.2	61.6
Pennsylvania	10,369	8,413	−18.9	79.7	64.7	−15.0
Rhode Island	1,071	2,326	117.2	97.6	212.0	114.3
Vermont	911	1,312	44.0	141.6	204.0	62.4
Virginia	1,332	1,403	5.3	15.4	16.3	0.8
West Virginia	735	2,470	236.1	41.0	137.7	96.7
Wisconsin	2,349	5,208	121.7	39.9	88.4	48.5
**Subtotal**	**34,557**	**59,734**	**72.9**	43.3	68.3	25.0
Low-incidence jurisdictions*						
Alabama	48	32	−33.3	1.0	0.6	−0.3
Alaska	8	7	−12.5	1.1	1.0	−0.1
Arizona	15	9	−40.0	0.2	0.1	−0.1
Arkansas	9	2	−77.8	0.3	0.1	−0.2
California	131	77	−41.2	0.3	0.2	−0.1
Colorado	5	10	100.0	0.1	0.2	0.1
Florida	180	233	29.4	0.8	1.1	0.2
Georgia	15	31	106.7	0.1	0.3	0.2
Hawaii	NR	NR	—	NR	NR	—
Idaho	14	10	−28.6	0.8	0.5	−0.2
Illinois	315	259	−17.8	2.5	2.0	−0.4
Indiana	162	236	45.7	2.4	3.5	1.0
Iowa	280	154	−45.0	8.8	4.8	−4.0
Kansas	35	9	−74.3	1.2	0.3	−0.9
Kentucky	21	72	242.9	0.5	1.6	1.1
Louisiana	8	5	−37.5	0.2	0.1	−0.1
Michigan	322	557	73.0	3.2	5.5	2.3
Mississippi	3	3	0	0.1	0.1	0
Missouri	13	7	−46.2	0.2	0.1	−0.1
Montana	9	13	44.4	0.8	1.2	0.4
Nebraska	13	9	−30.8	0.7	0.5	−0.2
Nevada	16	10	−37.5	0.5	0.3	−0.2
New Mexico	4	3	−25.0	0.2	0.1	−0.1
North Carolina	280	279	−0.4	2.7	2.7	0
North Dakota	42	22	−47.6	5.4	2.8	−2.6
Ohio	343	553	61.2	2.9	4.7	1.8
Oklahoma	0	0	0	0	0	0
Oregon	73	61	−16.4	1.7	1.4	−0.3
South Carolina	36	44	22.2	0.7	0.9	0.2
South Dakota	10	12	20.0	1.1	1.4	0.3
Tennessee	40	32	−20.0	0.6	0.5	−0.1
Texas	49	23	−53.1	0.2	0.1	−0.1
Utah	24	16	−33.3	0.7	0.5	−0.2
Washington	33	23	−30.3	0.4	0.3	−0.1
Wyoming	3	4	33.3	0.5	0.7	0.2
**Subtotal**	**2,561**	**2,817**	**10.0**	0.7	0.5	−0.2
**U.S. total**	**37,118** ^§§^	**62,551**	**68.5**	11.2	18.9	7.7

**Abbreviation**: NR = not reportable.

* High-incidence jurisdictions are defined as jurisdictions reporting 10 or more confirmed cases per 100,000 population for 3 years. All other jurisdictions are low incidence.

^†^ Lyme disease surveillance case definitions are available at https://ndc.services.cdc.gov/conditions/lyme-disease/. Case counts reflect the total number of cases (confirmed and probable).

^§^ Incidence is defined as the number of cases per 100,000 population according to 2020 U.S. Census Bureau data. Subtotal incidence figures reflect median incidence across jurisdictions in each incidence category.

^¶^ Cases and incidence during 2017–2019 reflect the 3-year annual average.

** Percent change in the number of cases reported during 2022 versus 2017–2019.

^††^ Incidence difference = (incidence in 2022 – 3-year average incidence during 2017–2019).

^§§^ Because of rounding of the average number of cases per jurisdiction, the total in the individual jurisdiction rows does not sum to the national 2017–2019 average.

### Sex and Age

**FIGURE 1:**
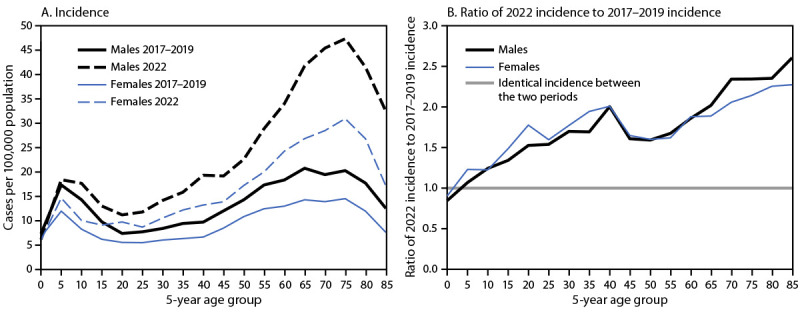
Reported Lyme disease incidence (A) and the ratio of the 2022 incidence to the average 2017–2019 incidence (B), by sex and 5-year age group — United States, 2017–2019 and 2022

Males accounted for the majority of cases during 2017–2019 (57.7%) and 2022 (57.3%). The age distribution was bimodal during both periods, but a larger percentage of reported cases occurred among adults in 2022 than did during 2017–2019 (Figure 1). Among persons aged 5–9 years, incidence during 2022 (16.5 cases per 100,000) was 11.5% higher than the 2017–2019 average (14.8). Among adults aged 75–79 years, incidence during 2022 (38.3) was 2.2 times the average during 2017–2019 (17.3) (Figure 1).

### Illness Onset and Other Available Dates

**FIGURE 2:**
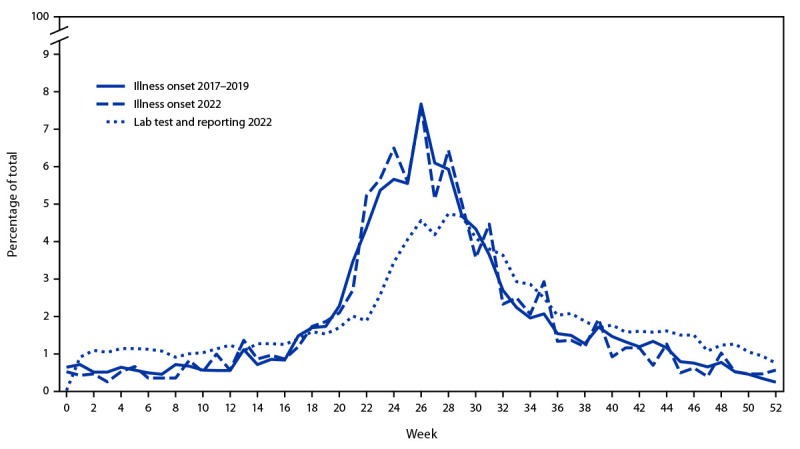
Week of illness onset or laboratory test and reporting date for reported Lyme disease cases* — United States, 2017–2019 and 2022 * Week 1 begins on the first Sunday of the calendar year.

Illness onset date was available for more than two thirds (67.8% [75,491 of 111,354]) of cases reported during 2017–2019, but only 4.8% (2,987 of 62,551) of cases in 2022. Illness onset peaked during calendar week 26 during both 2017–2019 and 2022; however, in 2022, the diagnosis, laboratory test, and reporting dates peaked 2 weeks later (week 28) (Figure 2).

## Discussion

After implementation of a revised surveillance case definition in 2022, the number of reported Lyme disease cases in the United States increased 68.5% over the average reported during 2017–2019; in high-incidence jurisdictions, the number of cases increased 72.9%, whereas in low-incidence jurisdictions, the number of cases increased 10.0%. This change reflects a large increase in the number of cases reported from high-incidence jurisdictions on the basis of laboratory evidence alone. Before 2022, many of these cases would have been excluded, either because health departments were unable to obtain the necessary clinical information or because available clinical data were inconsistent with the objective criteria specified in the case definition. The increases in incidence in 2022 compared with 2017–2019 are particularly large among high-incidence jurisdictions that had previously modified Lyme disease surveillance practice to minimize the case investigation workload. The total number of cases in many low-incidence jurisdictions decreased, presumably because of changes in the 2022 case definition requiring objective signs and symptoms of Lyme disease for the probable case classification in these areas with lower disease risk.

The relative increase in Lyme disease incidence in 2022 was larger among older age groups, with age-specific incidences more than doubling among adults aged ≥65 years relative to those during 2017–2019. The differential increase in incidence might reflect 1) more frequent laboratory testing among older age groups, 2) proportionally more disseminated illness in older age groups, and 3) proportionally more positive laboratory test results related to previous exposure to *B. burgdorferi* rather than a current illness.

Date of illness onset is rarely available in high-incidence jurisdictions given reliance on laboratory-based reporting without case investigation to ascertain clinical information. Alternative dates related to laboratory testing or reporting still demonstrate summer seasonality, but are shifted 2 weeks later, reflecting the expected time lag required after symptom onset to mount a detectable immune response to *B. burgdorferi* (*1*).

### Limitations

The findings in this report are subject to at least two limitations. First, surveillance for Lyme disease is subject to under- and overreporting. Despite an increase in reported cases in 2022, it is likely that current surveillance does not capture all cases of Lyme disease, specifically cases of early disease for which diagnosis is based on clinical findings alone, including presence of erythema migrans rash, and laboratory evidence is lacking because of insufficient elapsed time to mount a detectable antibody response. Previous case definitions relied on direct clinician report to identify such cases; however, the frequency of such reporting was highly variable among high-incidence jurisdictions (*6*). Conversely, reporting based solely on serologic testing might result in the inclusion of clinically incompatible or nonincident cases (i.e., a positive laboratory test result based on previous infection). Antibody titers remain elevated for months to years after treatment for Lyme disease, and asymptomatic seroconversion is also known to occur (*1*). In these instances, testing for Lyme disease when another etiology is responsible for the current illness might generate an erroneous case report. Second, changes in laboratory testing between the two analysis periods might have influenced Lyme disease incidence. The Food and Drug Administration cleared the first modified two-tier test (MTTT) serologic assays for Lyme disease in 2019^§§^ (*9*). These assays have higher sensitivity in early illness than do standard algorithms and might have resulted in more persons with positive laboratory evidence of infection (*10*). In contrast, health departments anecdotally reported challenges in receiving or identifying MTTT assays within their systems because of lack of MTTT-specific Logical Observation and Identifiers Names and Codes (LOINC), which might have resulted in underascertainment of persons with positive laboratory evidence in 2022.

### Implications for Public Health Practice

The 69% increase in reported cases of Lyme disease after implementation of the 2022 surveillance case definition, with the largest relative increase occurring among older adults, likely reflects modification of surveillance methods in high-incidence jurisdictions rather than a true change in disease risk. Surveillance in low-incidence jurisdictions still necessitates clinical investigation to ascertain probability of locally acquired infection to accurately guide clinical and public education. The revised approach to surveillance will improve standardization of surveillance data across high-incidence jurisdictions but precludes robust comparison of trends with data collected using earlier case definitions. Specific LOINC codes were created and approved in early 2023.^¶¶^ Use of standardized codes by commercial and clinical laboratories is critical to ensuring consistent identification of persons with laboratory evidence of Lyme disease for surveillance purposes. Although the total number of reported cases is higher than in previous years, it still does not approach the estimated 476,000 Lyme disease diagnoses estimated to occur annually in the United States (*2*), a frequency that highlights the need for effective prevention methods.

## Footnotes

* As of 2022, high-incidence jurisdictions are Connecticut, Delaware, District of Columbia, Maine, Maryland, Massachusetts, Minnesota, New Hampshire, New Jersey, New York, Pennsylvania, Rhode Island, Vermont, Virginia, West Virginia, and Wisconsin.

†
https://ndc.services.cdc.gov/conditions/lyme-disease/

§
https://www.cdc.gov/nndss/index.html

¶
https://data.census.gov/table?q=Age+and+Sex&t=Populations+and+People&g=010XX00US&d=DEC+Demographic+and+Housing+Characteristics&tid=DECENNIALDHC2020.P12 ; https://data.census.gov/table?g=010XX00US$0400000&tid=PEPPOP2019.PEPANNRES

** 45 C.F.R. part 46.102(l)(2), 21 C.F.R. part 56; 42 U.S.C. Sect. 241(d); 5 U.S.C. Sect. 552a; 44 U.S.C. Sect. 3501 et seq.

†† 2022 data from the National Notifiable Diseases Surveillance System. Interim data as of February 13, 2023, before finalization and publication by CDC’s Office of Public Health Data, Surveillance, and Technology.

§§
https://www.aphl.org/aboutAPHL/publications/Documents/ID-2021-Lyme-Disease-Serologic-Testing-Reporting.pdf

¶¶
https://loinc.org/
